# The effect of socioeconomic status on three-year mortality after first-ever ischemic stroke in Nanjing, China

**DOI:** 10.1186/1471-2458-6-227

**Published:** 2006-09-11

**Authors:** Guangyi Zhou, Xinfeng Liu, Gelin Xu, Xifei Liu, Renliang Zhang, Wusheng Zhu

**Affiliations:** 1 Department of Neurology, Jinling Hospital, Nanjing University School of Medicine, Nanjing, Jiangsu Province, PR China

## Abstract

**Background:**

Low socioeconomic status (SES) is associated with increased mortality after stroke in developed countries. This study was performed to determine whether a similar association also exists in China.

**Methods:**

A total of 806 patients with first-ever ischemic stroke were enrolled in our study. From August 1999 to August 2005, the three-year all-cause mortality following the stroke was determined. Level of education, occupation, taxable income and housing space were used as indicators for SES. Stepwise univariate and multivariate COX proportional hazards models were used to study the association between the SES measures and the three-year mortality.

**Results:**

Our analyses confirmed that occupation, taxable income and housing space were significantly associated with three-year mortality after first-ever stroke. Manual workers had a significant hazard ratio of 5.44 (95% CI 2.75 to 10.77) for death within three years when compared with non-manual workers. Those in the zero income group had a significant hazard ratio of 5.35 (95% CI 2.95 to 9.70) and those in the intermediate income group 2.10 (95% CI 1.24 to 3.58) when compared with those in the highest income group. Those in two of the three groups with the smallest housing space also had significant hazard ratios of 2.06 (95% CI 1.16 to 3.65) and 1.68 (95% CI 1.12 to 2.52) when compared with those in group with the largest housing space. These hazard ratios remained largely unchanged after multivariate adjustment for age, gender, baseline cardiovascular disease risk factors, and stroke severity. The analyses did not confirm an association with educational level.

**Conclusion:**

Lower SES has a negative impact on the outcome of first-ever stroke in Nanjing, China. This confirms the need to improve preventive and secondary care for stroke among low SES groups.

## Background

Because of different lifestyle and dietary patterns, the incidence of intracranial hemorrhage in China was substantially higher than in Western countries in the past [1,2]. With economic reform there has been a great improvement of the SES in China, especially in gross national product, household income and personal expenditures. And with the adoption of Western lifestyle and dietary habits (including increased energy intake, fat intake, alcohol consumption, cigarette smoking and decreased physical activity) by Chinese, the pattern of stroke has gradually shifted from hemorrhagic to ischemic [1]. Although ischemic stroke in China does not yet account for 80 percent of all stroke events as reported in Western populations [3], with the aging of the general population it is an increasingly important public health issue in China [1,4].

Previous studies have identified an association between low socioeconomic status (SES) and increased mortality after stroke [5-14]. Previous studies have also indicated that most patients were likely to die in the first year after stroke events and that their mortality would remain stable after that [15,16]. There has been a lack of detailed epidemiological information and few previous studies, however, to reflect if these conditions also exist in China. Thus, a cohort study based on the Nanjing Stroke Registry was undertaken to investigate the association between socioeconomic status and three-year mortality after ischemic stroke in China.

## Methods

### Subjects

The Nanjing Stroke Registry Program is the first hospital-based program designed to determine stroke incidence, risk factors, and prognosis in a multiethnic population in mainland China. The Program is based in Jinling Hospital, which serves the residents of Nanjing City. After obtaining informed consent, a total of 2200 patients with first-ever stroke were enrolled, retrospectively coded, and entered into a computerized data bank between August 1999 and August 2002. Patients with traumatic intracranial hemorrhages, brain tumors and subdural hematomas were not included. First-ever stroke was defined as a stroke occurring in a patient with no previous cerebral infarction or hemorrhage with neurological symptoms lasting greater than 24 hours, diagnosed by two qualified neurologists based on the World Health Organization (WHO) definition [17]. Because they have different etiological mechanisms and clinical manifestations, those with intracranial hemorrhages (n = 563) and subarachnoid hemorrhages (n = 61) were excluded. Those without CT or MRI scans (n = 770) were also excluded, since ischemic stroke and intracranial hemorrhages may be indistinguishable. The study therefore included 806 patients with first-ever ischemic strokes confirmed by CT or MRI scans. A structured interview was used to assess cerebrovascular disease risk factors. To guarantee the validity of the registration, all available information on every patient was rechecked weekly by an expert committee composed of neurologists and epidemiologists. The study was approved by the local ethics committee.

### Cerebrovascular disease risk factors

Cerebrovascular disease risk factors included age, sex, cigarette smoking, hypertension (i.e., blood pressure >140/90 mmHg at least twice before the stroke), diabetes mellitus, hypercholesterolemia (i.e., serum cholesterol concentration >6.5 mmol/L) on admission, atrial fibrillation (diagnosed and treated previously or during admission), myocardial infarction (diagnosed and treated previously or during admission), and prior transient ischemic attack (TIA). Smoking status was classified as current, former, or never. Current smoking was defined as one or more cigarettes daily at the onset of the stroke [19] and former smoking as smoking cessation at least one year before the onset of the stroke. Diabetes mellitus was defined as use of insulin or oral blood glucose-lowering drugs or repeat fasting blood glucose ≥ 7.0 mmol/L before the onset of the stroke. Heart disease was confirmed by 12-lead electrocardiogram and echocardiography. Histories of TIA were considered positive when confirmed by two neurologists. Stroke severity was documented with the NIH Stroke Scale (NIHSS) [18].

### Socioeconomic status

Information on SES was obtained through the systematic investigation of every patient. Level of education, occupation, taxable income and housing space were used as indicators of SES. According to a classification scheme used in China, the categories for education were as follows: (1) illiterate, (2) primary school, (3) junior high school, (4) senior high school, (5) technical training or apprenticeship, and (6) university or college degree. Participants were classified into three broad occupational classes: unemployed, manual workers, and non-manual workers. For each participant we obtained information on gross income and calculated the household income as the sum of the household members' gross income. The gross income included all income types subject to income taxation. The taxable gross income per month was categorized as the following: (1) 0 yuan, (2) > 0 yuan, <1000 yuan, (3) ≥ 1000 yuan. As for housing space, the habitable area per person in the household was determined and grouped as follows: (1) <10 m^2^/person, (2) ≥ 10 m^2^/person, <20 m^2^/person, (3) ≥ 20 m^2^/person, <40 m^2^/person, (4) ≥ 40 m^2^/person.

### Follow-up

For each case, the starting point of follow-up was the incident date of the stroke. The end point was the date of death from any cause within three years after the stroke. Patients who did not die during the three years after the stroke were censored. The latest data update on mortality was August 30, 2005. The low rate of out-migration after stroke (1.41%) and the unique household registry system in the Chinese urban community made it possible that the outcomes of these patients after first-ever stroke were readily determined. For the stroke patients who migrated out of the city, we obtained information about their survival status by telephone and mail. The follow-up was 100% complete.

### Statistical analysis

ANOVA tests, ***x***^**2 **^tests or Mann-Whitney U tests were used in the univariate comparisons in ordinal or continuous variables according to stroke outcome. On the basis of the methods described in previous articles [5,11], Spearman correlation analysis was undertaken to evaluate the association between measures of SES. Stepwise Cox proportional hazards models were used to study the association between each indicator of SES and three-year mortality. The first adjusted factors were age and sex, which have commonly been considered in previous studies as confounders in the association between SES indicators and health outcomes [20]. Second, we added smoking, an important behavior variable closely associated with stroke prognosis. Finally, all factors (including age, sex, smoking, hypertension, diabetes mellitus, hypercholesterolemia, atrial fibrillation, myocardial infarction, prior TIA, and NIHSS severity score) were considered. Probability of stepwise regression analysis for entry was defined as lower than 0.05. SPSS (version 13.0) was used for all analyses.

## Results

The mean age of the enrolled patients was 71.0 ± 11.2 years (median 71.0 years; range 23–100 years), 257(31.9%) of whom were women. During the follow-up period, 166 stroke patients had died, and the cumulative three-year mortality was 20.6%. The proportion of lower SES to higher SES was 1.02 at baseline.

Table 1 shows the baseline cerebrovascular disease risk factors and the SES factors for those who died and those who survived. The mean age of those in the death group was 7.8 years higher than that of those in the survival group (*p *< 0.001). The prevalence of cerebrovascular disease risk factors (e.g., hypertension, diabetes mellitus, hypercholesterolemia, atrial fibrillation, myocardial infarction) in the death group was significantly higher than in survival group (*p *< 0.01). Lower SES factors were more prevalent in the death group than in the survival group, which reached statistical significance (*p *< 0.001). The mean NIHSS score of the death group was also significantly higher than that of the survival group (*p *< 0.001), indicating more severe strokes in the death group. There was no difference in the prevalence of smoking or the prevalence of TIA history between the two groups.

Education was significantly associated with occupation, income, housing space (*r*^2 ^= 59.8%, *r*^2 ^= 66.5%, *r*^2 ^= 16.8%, respectively). This association also persisted between occupation and income (*r*^2 ^= 59.4%). Housing space strongly correlated with income (*r*^2 ^= 21.5%) but not with occupation (*r*^2 ^= 6.9%). Correlation was significant at *p *< 0.001.

Hazard ratios of death after first-ever stroke according to SES factors at different stages of correction for baseline factors are shown in Tables 2 and 3. Correlations between most predictors of SES (except educational level) and all-cause mortality were significant in both the univariate and multivariate models.

The univariate analysis showed that manual workers had a significantly increased risk of death after first-ever stroke when compared with non-manual workers, with a hazard ratio of 5.44 (95% CI 2.75 to 10.77, *p *< 0.001). The result remained largely unchanged after adjustment for the different baseline variables.

In the univariate model, there were hazard ratios of 5.35 (95% CI 2.95 to 9.70, *p *< 0.001) for those in the group with the lowest incomes and 2.10 (95% CI 1.24 to 3.58, *p *= 0.006) for those in the group with the second lowest incomes compared with those with the highest incomes. Furthermore, the ratio for the second lowest income group rose to 2.22 (95% CI 1.34 to 3.66, *p *= 0.002) in the last multivariate model.

A hazard ratio of 2.06 (95% CI 1.16 to 3.65, *p *= 0.013) for the group with the lowest level of housing space was also observed compared with those with the highest level, which rose to 2.13 (95% CI 1.23 to 3.70, *p *= 0.007) after adjustment for age, sex and cerebrovascular disease risk factors. Those in the group with the second lowest level of housing space group had a hazard ratio of 1.34 (95% CI 0.89 to 2.03) compared with those in the highest level, which failed to reach statistical significance.

## Discussion

The effect of SES on the risk of stroke and stroke-related mortality is an important public issue in China as well as in other countries. To the best of our knowledge, the present study was the first detailed assessment of this issue undertaken in China. Acknowledging the wide range of economic and educational diversity in China, a unique feature of our study was our focus on SES factors that reflect the status of individual patients, such as their occupation, income, housing space, and level of education. Our results showed that factors indicating a lower SES significantly correlate with greater three-year mortality after first-ever stroke. We also assessed our patients' risk factors for cerebrovascular disease, and our results showed there was a greater prevalence of risk factors (except smoking status and history of TIA) in those who did not survive three years after their strokes.

Overall, our findings were consistent with several previous studies. Arrich et al. [5] found that lower occupational status and lower income were associated with reduced survival of patients with acute stroke after adjustment for confounders, but they did not find an association with education level. Kapral et al. [11] demonstrated that both 30-day and 1-year mortality were higher in stroke patients in the lowest income quintile than those in the highest quintile. Jakovljevic et al. [10] described that their low-income group had a significantly greater mortality after first stroke than did their high-income group, both at day 28 and at one year. They also found that lower educational attainment was not significantly associated with greater mortality, except among women aged 25 to 59 years. Gillum and Mussolino [21] reported greater mortality after stroke in those with less than eight years of education and those in the lowest quartiles of their poverty index. Bennett [8] found that men in manual occupations were at least 60 percent more likely to die from stroke than men in professional occupations. On the other hand, there have been studies that have reported either no association or a weak association between SES and survival [22-24]. Two probable explanations for these differences are the disparities between the countries in the definition of SES and the inconsistency of the data sources (e.g., hospital-based versus population-based).

There are different hypothetical mechanisms that may explain our findings. Differences in occupations may reflect differences in factors such as the physical and psychosocial dimensions of work [25]. As Lynch and colleagues [26] have proposed, a combination of higher individual income and better physical and social infrastructure has the potential to reduce health inequalities and improve public health. For example, in the center of Nanjing City, many low-income patients can only afford homes in poorly managed, lower priced housing, and many are even homeless. They are therefore constantly exposed to environmental toxins and hazards and are subjected to increased psychological stress and poor health care, all of which jeopardize their health [27]. Interestingly, previous studies have not emphasized housing space, and our finding that less housing space was associated with increased three-year mortality after stroke strongly supports this hypothesis. Higher patient education may be a marker of better material and psychosocial conditions, including better diet, less stress, access to preventive care, and increased receptivity to messages that promote health [21], but perhaps due to cultural differences, we found only a weak association between lower education and greater mortality in this study.

Several limitations of this study should be mentioned. First, because this study is hospital-based, we acknowledge the possibility of selection bias. Our results, for example, may not reflect the overall conditions in China because of geographical differences, since the population-based Sino-MONICA project [4] showed that the mortality associated with stroke in China was higher in the north compared to the south. The confined sample size in our hospital-based study also means that our results may not accurately reflect the overall association between SES and stroke mortality in China. Second, in traditional Chinese society, the elderly are cared for by their offspring, and consequently many patients with relatively minor strokes may not have been hospitalized. In addition, stroke patients in rural areas may have been misdiagnosed or not treated according to modern standards by local doctors practicing traditional Chinese medicine. Also due to cultural differences, many stroke patients may have died at home without ever seeking medical attention. Many stroke patients, therefore, may not have been enrolled in our stroke registry, and although applicable to Nanjing, our findings may not be representative of the Chinese population in general.

## Conclusion

Despite its limitations, the present study provides detailed information about the impact of SES on the three-year mortality of patients hospitalized for first-ever stroke in China. In order to improve the outcome after stroke for all patients worldwide, large-scale, long-term studies should be undertaken to clarify the mechanisms underlying SES and stroke mortality.

## Competing interests

The author(s) declare that they have no competing interests.

## Authors' contributions

GZ contributed to study design, data collection, data analysis and paper writing. XL (Xinfeng) participated in study design, data analysis and revised the paper. GX participated in data collection, data analysis and paper writing. XL (Xifei) and RZ contributed to data collection and paper writing. WZ participated in data collection and data analysis. All authors reviewed drafts of the manuscript and approved the version to be published.

## Pre-publication history

The pre-publication history for this paper can be accessed here:



## Figures and Tables

**Table 1:** Baseline Characteristics of Stroke Patients According to Stroke Outcome

	Death (n = 166)	Survival (n = 640)	*p*
			
	No. (%)	No. (%)	
Mean age, y	77.2 ± 8.5	69.4 ± 11.3	<0.001*
Female	75(45.2)	182(28.4)	<0.001
Current smoker	46(27.7)	141(22.0)	0.076
SBP (mmHg)	175.4 ± 4.6	143.5 ± 2.8	<0.001*
Diabetes mellitus	44(26.5)	112(17.5)	0.007
Hypercholesterolemia	54(32.5)	112(17.5)	<0.001
Atrial fibrillation	112(67.5)	32(5.0)	<0.001
Myocardial infarction	25(15.1)	31(4.8)	<0.001
Prior TIA	24(14.5)	115(18.0)	0.286
NIHSS mean (IQR)	6.3(1–9)	5.3(1–9)	<0.001†
Education			
Illiteracy	58(34.9)	56(8.8)	<0.001
Primary school	55(33.1)	84(13.1)	<0.001
Junior high school	26(15.7)	188(29.4)	<0.001
Senior high school	8(4.8)	95(14.8)	0.001
Technical training	6(3.6)	61(9.5)	0.014
University degree	13(7.8)	156(24.4)	<0.001
Occupation			
Unemployed	23(13.9)	18(2.8)	<0.001
Manual worker	127(76.5)	256(40.0)	<0.001
Non-manual worker	16(9.6)	366(57.2)	<0.001
Income			
0 yuan	63(38.0)	27(5.8)	<0.001
<1000 yuan	75(45.2)	185(28.9)	<0.001
≥ 1000 yuan	28(16.9)	428(66.9)	<0.001
Housing space			
< 10 m^2^/person	17(10.2)	6(0.9)	<0.001
10 to 20 m^2^/person	44(26.5)	87(13.6)	<0.001
20 to 40 m^2^/person	42(25.3)	197(30.8)	0.168
≥ 40 m^2^/person	63(38.0)	350(54.7)	<0.001

*p *values are based on ***x***^**2 **^test.

* *p *values are based on ANNOVA test.

† *p *values are based on Mann-Whitney U test.

**Table 2:** Hazard Ratios (95% CI) for Categories of Socioeconomic Status at the first and second Stage of Correction for Baseline Factors

	Univariate	*p*	Plus age and sex	*p*
Education				
Illiteracy	1.04(0.50–2.18)	0.913	0.96(0.46–2.00)	0.916
Primary school	1.37(0.63–2.98)	0.423	1.25(0.57–2.72)	0.579
Junior high school	0.58(0.26–1.30)	0.184	0.77(0.34–1.74)	0.533
Senior high school	0.73(0.28–1.89)	0.520	1.05(0.41–2.71)	0.923
Technical training	1.49(0.54–4.14)	0.447	1.99(0.71–5.61)	0.192
University degree	1		1	
Occupation				
Unemployed	7.03(3.27–15.10)	<0.001	6.27(2.97–13.21)	<0.001
Manual worker	5.44(2.75–10.77)	<0.001	5.23(2.88–9.49)	<0.001
Non-manual worker	1		1	
Income				
0 yuan	5.35(2.95–9.70)	<0.001	5.37(3.19–9.03)	<0.001
<1000 yuan	2.10(1.24–3.58)	0.006	2.22(1.36–3.63)	0.002
≥ 1000 yuan	1		1	
Housing space				
< 10 m^2^/person	2.06(1.16–3.65)	0.013	2.07(1.19–3.59)	0.010
10 to 20 m^2^/person	1.34(0.89–2.03)	0.159	1.38(0.92–2.07)	0.115
20 to 40 m^2^/person	1.68(1.12–2.52)	0.012	1.61(1.07–2.41)	0.021
≥ 40 m^2^/person	1		1	

For each variable, the last category serves as reference category.

**Table 3:** Hazard Ratios (95% CI) for Categories of Socioeconomic Status at the third and last Stage of Correction for Baseline Factors

	Plus smoking	*p*	Plus all factors*	*p*
Education				
Illiteracy	0.76(0.35–1.67)	0.492	0.79(0.31–2.01)	0.624
Primary school	1.11(0.50–2.48)	0.801	0.97(0.39–2.40)	0.940
Junior high school	0.80(0.36–1.80)	0.592	0.70(0.28–1.77)	0.455
Senior high school	1.16(0.45–3.02)	0.757	0.96(0.33–2.78)	0.934
Technical training	1.98(0.70–5.60)	0.197	1.81(0.62–5.28)	0.277
University degree	1		1	
Occupation				
Unemployed	6.85(3.21–14.60)	<0.001	5.77(2.67–12.46)	<0.001
Manual worker	5.23(2.87–9.52)	<0.001	5.18(2.84–9.44)	<0.001
Non-manual worker	1		1	
Income				
0 yuan	5.94(3.49–10.11)	<0.001	5.45(3.18–9.34)	<0.001
<1000 yuan	2.34(1.43–3.84)	0.001	2.22(1.34–3.66)	0.002
≥ 1000 yuan	1		1	
Housing space				
< 10 m^2^/person	2.11(1.22–3.66)	<0.001	2.13(1.23–3.70)	0.007
10 to 20 m^2^/person	1.29(0.86–1.93)	0.223	1.17(0.77–1.77)	0.473
20 to 40 m^2^/person	1.66(1.11–2.49)	0.013	1.52(1.01–2.28)	0.046
≥ 40 m^2^/person	1		1	

For each variable, the last category serves as reference category.

* All factors include including age, sex, smoking, hypertension, diabetes mellitus, hypercholesterolemia, atrial fibrillation, myocardial infarction, prior TIA and NIHSS.
